# Prognostic role of serum albumin levels in patients with chronic heart failure

**DOI:** 10.1007/s11739-024-03612-9

**Published:** 2024-05-22

**Authors:** Giuseppe Armentaro, Valentino Condoleo, Carlo Alberto Pastura, Maria Grasso, Angelo Frasca, Domenico Martire, Velia Cassano, Raffaele Maio, Leonilde Bonfrate, Daniele Pastori, Tiziana Montalcini, Francesco Andreozzi, Giorgio Sesti, Francesco Violi, Angela Sciacqua

**Affiliations:** 1 Department of Medical and Surgical Sciences, University Magna Graecia of Catanzaro, 88100 Catanzaro, Italy; 2 Section of Gastroenterology and Hepatology, Department of Health Promotion, Mother and Child Care, Internal Medicine and Medical Specialties (PROMISE), University of Palermo, Piazza Delle Cliniche N.2, 90127 Palermo, Italy; 3 Clinica Medica “A. Murri”, Department of Biomedical Sciences and Human Oncology, University of Bari Aldo Moro, Bari, Italy; 4 Department of Clinical Internal, Anesthesiological and Cardiovascular Sciences, Sapienza University of Rome, Viale del Policlinico 155, 00161 Rome, Italy; 5 Department of Clinical and Experimental Medicine, University Magna Grecia, 88100 Catanzaro, Italy; 6 Research Center for the Prevention and Treatment of Metabolic Diseases (CR-METDIS), University Magna Græcia, 88100 Catanzaro, Italy; 7 Department of Clinical and Molecular Medicine, University Rome-Sapienza, Viale Regina Elena N. 324, 00161 Rome, Italy

**Keywords:** Chronic heart failure, Albumin, MACE, Comorbidities

## Abstract

**Background:**

Hypoalbuminemia is common in heart failure (HF) patients; however, there are no data regarding the possible long-term prognostic role of serum albumin (SA) in the younger population with chronic HF without malnutrition. The aim of this study was to examine the long-term prognostic role of SA levels in predicting major adverse cardiac events (MACE) in middle-aged outpatients with chronic HF.

**Methods:**

In the present retrospective analysis, 378 subjects with HF were enrolled. MACE (non-fatal ischemic stroke, non-fatal myocardial infarction, cardiac revascularization or coronary bypass surgery, and cardiovascular death), total mortality, and HF hospitalizations (hHF) occurrence were evaluated during a median follow-up of 6.1 years.

**Results:**

In all population, 152 patients had a SA value < 3.5 g/dL and 226 had a SA value ≥ 3.5 g/dL. In patients with SA ≥ 3.5 g/dL, the observed MACE were 2.1 events/100 patient-year; while in the group with a worse SA levels, there were 7.0 events/100 patient-year (*p* < 0.001). The multivariate analysis model confirmed that low levels of SA increase the risk of MACE by a factor of 3.1. In addition, the presence of ischemic heart disease, serum uric acid levels > 6.0 mg/dL, chronic kidney disease, and a 10-year age rise, increased the risk of MACE in study participants. Finally, patients with SA < 3.5 g/dl had a higher incidence of hHF (*p* < 0.001) and total mortality (*p* < 0.001) than patients with SA ≥ 3.5 g/dl.

**Conclusions:**

Patients with chronic HF that exhibits low SA levels show a higher risk of MACE, hHF and total mortality.

**Supplementary Information:**

The online version contains supplementary material available at 10.1007/s11739-024-03612-9.

## Introduction

Heart failure (HF) is a growing health problem, prevalence ranges from about 1.5 to 4% in developed countries [1]. Despite important pharmacological innovations in recent years, the prognosis of HF patients remains poor with a mortality rate of about 50% during the five-year follow-up [1]. Another critical issue in HF is represented by comorbidities that show a crucial role on diagnosis, treatment, and prognosis. Among non-cardiovascular (CV) comorbidities malnutrition and frailty, often under-diagnosed and undertreated, have particular relevance affecting clinical prognosis especially in elderly [2–4].

In this setting, hypoalbuminemia represents a marker of malnutrition and frailty, but also of comorbidities and inflammatory state burden [5, 6]. Hypoalbuminemia is a common condition in HF, with a prevalence up to 89% and is associated with an increased risk of HF onset and progression, as well as CV morbidity and mortality [6–15]. These findings are most evident in acute HF also in nonagenarian patients, in which severe hypoalbuminemia is a potential predictor of poor in-hospital outcome [16–18]. In addition, hypoalbuminemia was found to be a significant independent predictor of mortality in both acute and chronic HF; however, the follow-up in these studies rarely exceeded one year and study populations are mainly represented by elderly with acute HF [17–27].

The prognostic role of albumin is also confirmed in chronic HF patients with secondary mitral insufficiency [28]. Furthermore, recent studies suggest that changes in SA levels over time are associated with different prognosis in patients with chronic HF. In fact, while a decrease in SA levels over time is associated with a worse prognosis, an increase is associated with a better prognosis; this is probably because SA levels reflect the general condition of chronic HF patients [29]. Data are also confirmed by a recent meta-analysis, where hypoalbuminemia worsens the prognosis in both inpatients and outpatients [30].

In addition, the prognostic value of SA is particularly evident in high-intensity care populations such as HF patients in cardiogenic shock or patients in intensive care; indeed, in this category of patients, in addition to reduced SA levels, a reduction in SA levels during hospitalization worsens the prognosis [31, 32]. To our knowledge, the prognostic significance of hypoalbuminemia has not yet been fully evaluated in the HF clinical setting. According with this, there are no data regarding the possible long-term prognostic role of albumin in the younger population with chronic HF, especially in patients without malnutrition. The purpose of the present study was to examine the long-term prognostic role of serum albumin (SA) levels in predicting major adverse cardiac events (MACE) in middle-aged outpatients with chronic HF, after adjustment for known confounding factors and especially for inflammation, nutritional status, liver and kidney function. Total mortality and HF hospitalizations (hHF) during follow-up were also evaluated.

## Materials and methods

### Study population

In this retrospective analysis, 378 subjects with HF were enrolled, between October 2012 and June 2022, at the Geriatric Department of “Magna Graecia” University-Hospital of Catanzaro, Italy. The study included outpatients suffering from chronic HF from the CATAnzaro Metabolic Risk factors (CATAMERI) Study, an ongoing longitudinal observational study assessing cardio-metabolic risk in individuals, recruited at the University Hospital of Catanzaro. They were recruited according to the indications of the European Society of Cardiology (ESC) guidelines for the diagnosis and treatment of acute and chronic HF [1]. The eligibility criteria included: written informed consent; age > 18 years; NYHA classes II–III. The exclusion criteria included: acute HF in the previous 6 months, chronic HF with NYHA class IV, respiratory failure, severe renal dysfunction (estimated glomerular filtrate (eGFR) < 30 mL/min/1.73m2); nephrotic syndrome, macroalbuminuria, severe hepatic impairment (Child–Pugh Class C); pregnancy or breastfeeding, malnutrition evaluated as Mini Nutritional Assessment (MNA) < 17 pts and cachexia [33]. A careful medical history was obtained in all subjects. A complete CV physical examination was performed, and both body weight and body mass index (BMI) were also measured. Blood samples were collected for the determination of several laboratory parameters.

The study was approved by the local Institutional Ethics Committees of University “Magna Graecia” of Catanzaro (code protocol number 2012.63). All patients signed informed consent and the study procedures were carried out in accordance with the principles of the Declaration of Helsinki.

### Laboratory parameters

All laboratory measurements were performed on peripheral blood samples after at least 12 h of fasting. SA was measured with a colorimetric spectrophotometric method (Bromocresol green). Glycaemia was determined by the glucose oxidase method (glucose analyzer, BeckmanCoulter, Milan) and the homeostasis model assessment (HOMA) index was used for the determination of Insulin resistance [34]. Enzymatic methods (Roche Diagnostics GmbH, Mannheim, Germany) were used for determination of blood levels of total cholesterol, low-density lipoprotein (LDL) cholesterol, high-density lipoprotein (HDL) cholesterol, and triglycerides. Alanine aminotransferase (ALT), aspartate aminotransferase (AST) by pyridoxal phosphate activated (liquid reagent), and gamma-glutamyltransferase (γ-GT) were evaluated by standardized method (COBAS Integra 800-Roche Diagnostics GmbH, Mannheim, Germany). Creatinine was measured using the Jaffé method. The CKD-EPI (Chronic Kidney Disease Epidemiology Collaboration) equation was used for the estimation of glomerular filtration rate (eGFR) [35]. Serum uric acid (UA) levels were assessed using URICASE/POD method (Boehringer Mannheim, Mannheim, Germany). The immuno-turbidimetric method automated system (Cardio Phase hs-CRP, Milan, Italy) was used to assess the high-sensitivity C-reactive protein (hs-CRP).

### Cardiovascular endpoints

MACE were identified as study endpoints (non-fatal ischemic stroke, non-fatal coronary events, and CV death). Total mortality and HF hospitalizations (hHF) during follow-up were also evaluated. The diagnosis of myocardial infarction was made according to the universal definition [36]. CV death included progressive HF and death related to surgical or percutaneous revascularization procedures, and sudden death. The diagnosis of ischemic stroke was determined by clinical manifestations and radiological findings [37]. Data on MACE were collected during the follow-up. A standardized form was filled out by the examiners when an event occurred. Details of each event were recorded, as well as death certificates, hospital discharge letter or copy of hospitalization medical record, and other clinical documentation obtained from patients or their relatives.

### Statistical analysis

Data were expressed as mean ± standard deviation (SD), median and interquartile range (IQR), and number and percentage for categorical variables, when appropriate. Student’s *t*-test was performed for unpaired data for continuous variables, Mann–Whitney’s test for unpaired data for non-continuous variables and *χ*^2^ tests for categorical variables. According to the clinical cut-off of SA (3.5 g/dL), the overall population was divided into two groups; of interest, this value has been already associated with increased thrombotic risk [38, 39]. Simple linear regression and multivariate stepwise linear regression models were constructed to evaluate independent predictors of hypoalbuminemia, both as a continuous and dichotomous variable. Variables that correlated significantly with MACE were entered into a multivariate stepwise linear regression model to evaluate independent predictors of SA levels. The accuracy of the SA value as a predictor of MACE, both as a categorical and continuous variable, was evaluated by processing a receiver operating characteristic (ROC) curve. The area under the curve (AUC) described the magnitude to which the SA value was associated with the onset of events. The number of events per 100 patient-year was used to calculate the incidence of events. The onset of MACE was not assessed at the same time, because the follow-up was not uniform for all patients, so a regression analysis based on the Cox proportional model was used, correcting the analysis for possible covariates associated with the finding of MACE. In particular, a univariate Cox regression model was performed on the incidence of MACE; subsequently, the variables that significantly correlated with the appearance of MACE were included in a multivariate Cox regression model to calculate the hazard ratio (HR) for the independent predictors associated with the incidence of MACE. Analysis was corrected for pharmacological treatments. The differences were considered statistically significant for p value < 0.05. All analyses were performed using the SPSS 20.0 statistical program for Windows (SPSS Inc., Chicago, IL, USA).

## Results

### Study population

378 patients were enrolled with average age of 67.1 ± 11.2 years, 107 were females and 271 males, 220 had HF with mildly reduced (HFmrEF) and preserved ejection fraction (HFpEF) and 158 were affected by HF with reduced ejection fraction (HFrEF). In all study population, 152 patients had a SA value < 3.5 g/dL, and none of these received albumin supplementation during follow-up, while the remaining 226 had a SA value ≥ 3.5 g/dL (Supplementary Fig. 1). The median follow-up was 6.1 (3.1–9.9) years.

Table 1 shows the epidemiological and clinical characteristics of the entire study population, stratified by the clinical cut-off of SA (3.5 g/dL). Statistically significant differences between the two groups were observed for prevalence of Non-alcoholic fatty liver disease (NAFLD) and dyslipidemia significantly higher in patients with lower SA levels. There was no difference between the two groups for intake of antihypertensive drugs, oral antidiabetics, insulin, oral anticoagulants, anti-aggregants, statins, beta-blockers, loop diuretics, SGLT2-inhibitors, sacubitril–valsartan, and mineralocorticoid receptor antagonists. Regarding clinical, hemodynamic, and laboratory parameters, there were no statistically significant differences between the two groups, except, obviously, for SA levels, but also for hs-CRP and microalbuminuria, significantly higher in patients with SA < 3.5 g/dL (Table 1). In addition, the two groups differed in MNA with worse results in lower albumin levels group.

**Table 1:** Clinical, epidemiological, laboratory, echocardiographic parameters, and pharmacotherapy of study population at baseline according to clinical cut-off of serum albumin

	All population (*n* 378)	Albumin < 3.5 g/dl (*n*. 152)	Albumin ≥ 3.5 g/dl (*n*. 226)	p^*^
Age, years	67.0 ± 11.2	67 ± 10.9	66.9 ± 11.4	0.551^ǂ^
Female gender, *n* (%)	107 (28.3)	46 (30.2)	61 (26.9)	0.488*
BMI, *Kg/m*^2^	31.0 ± 4.8	30.8 ± 4.6	31.1 ± 5.0	0.450^ǂ^
IHD, *n* (%)	233 (61.6)	95 (62.5)	138 (61.0)	0.778*
Arterial hypertension, *n *(%)	196 (51.9)	72 (47.4)	124 (54.9)	0.152*
AF, *n* (%)	76 (20.1)	26 (17.1)	50 (22.1)	0.232*
Dyslipidemia, *n* (%)	145 (38.9)	69 (45.4)	76 (33.6)	0.021*
SAS, *n* (%)	119 (31.4)	55 (36.1)	64 (28.3)	0.106*
CKD, *n* (%)	95 (21.1)	41 (26.9)	54 (23.9)	0.498*
COPD, *n* (%)	81 (21.4)	35 (23.0)	46 (20.3)	0.534*
NAFLD, *n* (%)	42 (11.1)	29 (19.0)	13 (5.7)	< 0.001*
Obesity, *n* (%)	92 (24.3)	41(26.9)	51(22.6)	0.327*
T2DM, *n* (%)	193 (51.0)	78 (51.3)	115 (50.1)	0.934*
Alcohol, *n* (%)	63 (16.6)	22 (14.4)	41 (18.1)	0.348*
Smokers, *n* (%)	134 (35.4)	59 (38.8)	75 (33.1)	0.261*
MLHFQ, *pt*	95.3 ± 11.2	95.23 ± 3.0	226 ± 3.1	0.619^ǂ^
MNA, *pts*	22.9 ± 3.3	21.7 ± 2.9	23.8 ± 3.2	< 0.001^ǂ^
SBP, *mmHg*	123.0 ± 15.6	122.0 ± 15.9	124.0 ± 15.4	0.166^ǂ^
DBP, *mmHg*	74.09 ± 9.7	73.9 ± 10.1	74.3 ± 9.4	0.704^ǂ^
HR, *bfm*	68.9 ± 14.5	69.7 ± 15.5	68.42 ± 13.9	0.387^ǂ^
RR, *afm*	18.9 ± 1.8	18.8 ± 1.9	18.9 ± 1.7	0.745^ǂ^
Hb, *g/dl*	12.2 ± 1.6	12.1 ± 1.7	12.2 ± 1.5	0.546^ǂ^
Hct, *%*	38.3 ± 5.4	37.9 ± 5.5	38.53 ± 5.3	0.304^ǂ^
Na, *mmol/l*	140.7 ± 33.1	140.61 ± 3.0	140.7 ± 2.5	0.685^ǂ^
Mg, *mg/dl*	1.9 ± 0.2	1.95 ± 0.2	1.93 ± 0.2	0.742^ǂ^
K, *mmol/l*	4.4 ± 0.41	4.4 ± 0.3	4.4 ± 0.4	0.503^ǂ^
HOMA, *pts*	6.0 ± 2.0	6.1 ± 2.9	5.9 ± 2.9	0.502^ǂ^
HbA1c, %	6.7 ± 0.7	6.7 ± 0.6	6.7 ± 0.7	0.827^ǂ^
eGFR, *ml/min/1.73m*^*2*^	70.7 ± 18.9	68.6 ± 18.5	72.2 ± 19.0	0.071^ǂ^
Microalbuminuria, *mg/l*	34.2 ± 14.2	41.4 ± 17.4	29.3 ± 8.6	< 0.001^ǂ^
LDL, *mg/dl*	63.36 ± 28.9	62.32 ± 27.2	64.06 ± 30.1	0.566^ǂ^
Triglycerides, *mg/dl*	128.0 ± 46.1	124.5 ± 34.8	131.6 ± 52.3	0.147^ǂ^
AST, *U/L*	21.4 ± 9.2	20.7 ± 7.9	21.9 ± 9.9	0.205^ǂ^
ALT, *U/L*	20.8 ± 9.7	19.6 ± 6.9	21.5 ± 11.2	0.063^ǂ^
γ-GT, *U/L*	36.0 ± 20.2	36.1 ± 18.4	35.9 ± 21.4	0.941^ǂ^
Albumin, *mg/dl*	3.8 ± 0.46	3.3 ± 0.2	4.1 ± 0.3	< 0.001^ǂ^
NT-pro-BNP, *pg/ml*	2283.3 ± 582.4	2320.8 ± 556.0	2258.1 ± 19.0	0.305^ǂ^
Uricemia, *mg/dl*	5.9 ± 1.2	4.5 ± 1.1	5.8 ± 1.3	0.173^ǂ^
hs-CRP, *mg/l*	3.9 ± 0.9	4.5 ± 1.1	3.4 ± 0.4	< 0.001^ǂ^
LAVi, *ml/m*^*2*^	44.5 ± 11.5	44.5 ± 12.4	44.5 ± 10.9	0.990^ǂ^
LVEF, %	43.0 ± 9.1	42.9 ± 9.3	43.1 ± 8.9	0.816^ǂ^
CI, *ml/min/1.73 m*^*2*^	1909.1 ± 197.6	1897.6 ± 196.1	1916.7 ± 198.6	0.358^ǂ^
E/A	0.80 ± 0.46	0.79 ± 0.47	0.81 ± 0.45	0.817^ǂ^
E/e’	15.4 ± 4.6	15.5 ± 4.6	15.3 ± 4.5	0.681^ǂ^
TAPSE, *mm*	17.6 ± 3.8	17.4 ± 3.5	17.7 ± 4.0	0.369^ǂ^
s-PAP, *mmHg*	42.3 ± 11.1	43.1 ± 10.1	41.7 ± 11.3	0.237^ǂ^
TAPSE/s-PAP, *mm/mmHg*	0.45 ± 0.19	0.44 ± 0.18	0.46 ± 0.19	0.199^ǂ^
β-blockers, *n* (%)	337 (89.1)	134 (77.9)	203 (89.8)	0.609*
ACEi/ARBs, *n* (%)	189 (50.0)	73 (48.0)	116 (51.3)	0.529*
MRAs, *n* (%)	156 (41.3)	60 (39.4)	96 (42.4)	0.560*
ARNI, *n* (%)	171 (45.2)	72 (47.4)	99 (43.8)	0.494*
SGLT2i, *n* (%)	158 (41.8)	66 (43.4)	92 (40.7)	0.600*
Loop diuretics, *n* (%)	297 (78.6)	127 (83.5)	170 (75.2)	0.053*
OADs, *n* (%)	193 (50.9)	78 (51.3)	115 (50.9)	0.934*

*performed by chi-square test

^ǂ^ performed by t-test for unpaired data

*BMI* body mass index, *IHD* ischemic heart disease, *AF* atrial fibrillation, *SAS* sleep apnea syndrome, *CKD* chronic kidney disease, *COPD* chronic obstructive pulmonary disease, *NAFLD* non-alcoholic fatty liver disease, *T2DM* type 2 diabetes mellitus, *MLHFQ* minnesota living with heart failure questionnaire, *MNA* mini-nutritional assessment, *SBP* systolic blood pressure, *DBP* diastolic blood pressure, *HR* heart rate, *RR* respiratory rate, *Hb* hemoglobin, *Hct* hematocrit, *Na* Sodium, *Mg* Magnesium, *K* Potassium, *HOMA* homeostatic HbA1c: glycated hemoglobin, *e-GFR* estimate glomerular filtration rate, *LDL* low-density lipoproteins, *AST* aspartate aminotransferase, *ALT* alanine aminotransferase, *γ-GT* gamma-glutamyltransferase, *NT-pro-BNP* N-terminal pro-brain natriuretic peptide, *hs-CRP* high-sensitivity C-reactive protein, *LAVi* left atrial volume index, *LVEF* left ventricular ejection fraction, *CI* cardiac index, *RVOTp* right ventricular outflow tract proximal, *E/A* ratio between wave E (the wave of rapid filling in early diastole) and wave A (the wave of atrial contraction), *E/e’* between wave E and wave e′ (reliable estimate of changes in end-diastolic blood pressure), *TAPSE* tricuspid annular plane systolic excursion, *s-PAP* systolic pulmonary arterial pressure, *ACEi* angiotensin-converting enzyme inhibitors, *ARBs* Angiotensin II receptor blockers, *MRAs* mineralocorticoid receptor antagonists, *ARNI* angiotensin receptor/neprilysin inhibitor, *SGLT2i* sodium–glucose cotransporter 2 inhibitors, *OADs* oral antidiabetic drugs

### Variables associated with hypoalbuminemia in the study population

From the first simple linear regression model, considering albumin as a continuous variable, the following variables correlated significantly: hs-CRP (*r* = −0.398; *p* < 0.001), MNA score < 23 pts (*r* = 0.189; *p* < 0.001), NAFLD (*r* = −0.206; *p* < 0.001) and microalbuminuria (*r* = −0.109; *p* = 0.015). At multivariate stepwise linear regression, the variable that was most associated with serum albumin levels were hs-CRP serum levels and the whole model correlated for 35.3% of albumin levels. Data were also confirmed by the second simple linear regression model; in fact, considering albumin as a dichotomous variable, the following variables correlated significantly: hs-CRP (*r*-0.513; *p* < 0.001), MNA score < 23 pts (*r* = 0.213; *p* < 0.001), NAFLD (*r* = −0.114; *p* = 0.004) and microalbuminuria (*r* = −0.039; *p* = 0.004). At multivariate stepwise linear regression analysis, hs-CRP levels were the main variable that was most associated, and the whole model correlated for 43.6% of SA levels (supplementary Tables 1–2).

### Major adverse cardiovascular events and hospitalization for Heart Failure

**Fig. 1:**
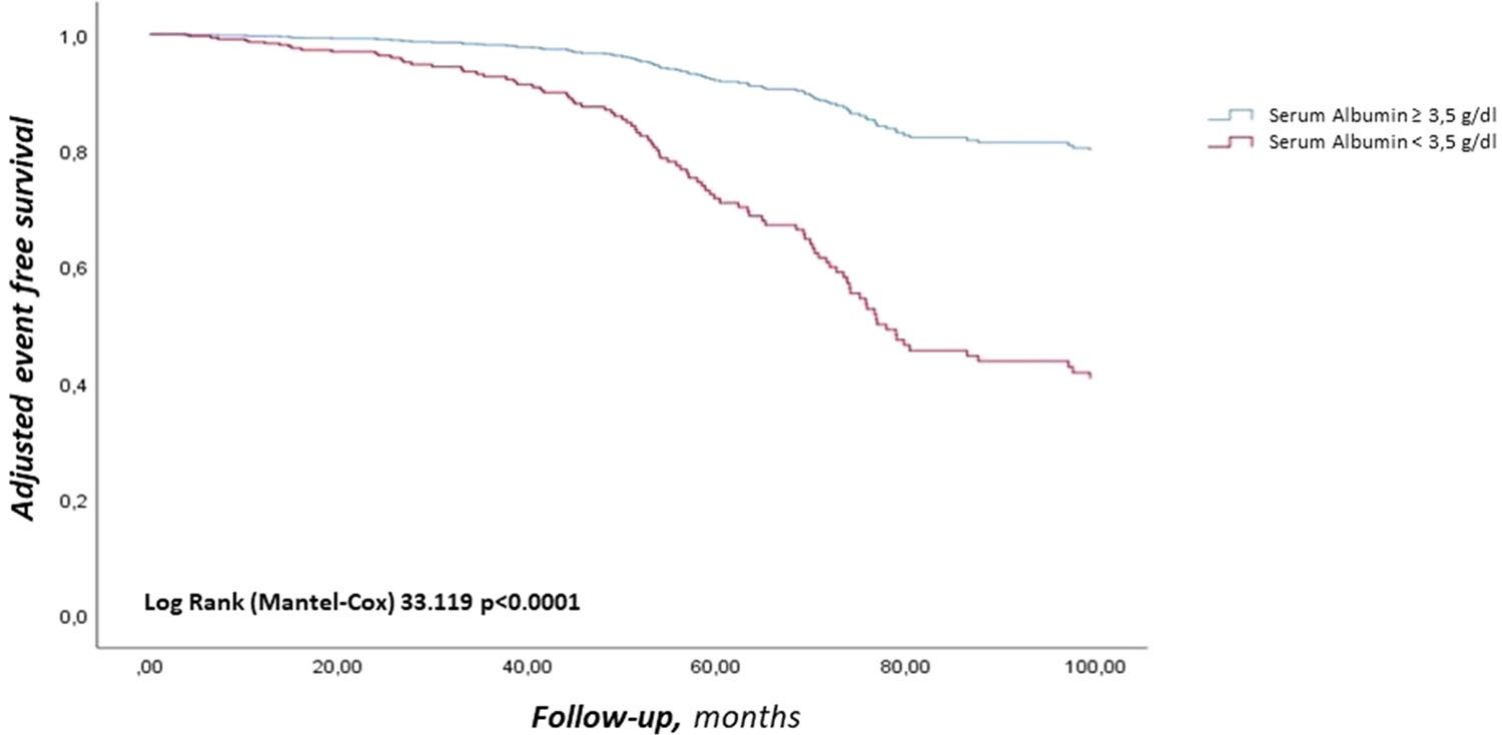
Adjusted Kaplan–Meier on MACE, according to cut-off value of SA, Adjusted for: SA as dichotomous value, IHD, UA as dichotomous value, CKD, and Age as 10 years. *MACE* major adverse cardiovascular events, *SA* Serum albumin, *IHD* ischemic heart disease, *UA* uric acid, *CKD* chronic kidney disease

In the whole population a total of 94 (24.9%) MACE were observed (3.8 events/100 patient-year) (Fig. 1). In particular, 45 (11.9%) were non-fatal coronary events (1.8 events/100 patient-year), 25 (6.6%) non-fatal stroke events (1.0 events/100 patient-year) and 24 (6.3%) CV death (0.9 events/100 patient-year). In patients with SA < 3.5 g/dL, MACE observed were 60 (6.8 events/100 patient-year); while in the group with SA ≥ 3.5 g/dL were 34 (2.1 events/100 patient-year) (*p* < 0.001) (Log-Rank test *p* < 0.001, Fig. 1). Non-fatal coronary events were 27 (17.8%) in the first group (3.1 events/100 patient-year) and 18 (7.9%) in the second group (1.1 events/100 patient-year) (*p* = 0.003); furthermore, non-fatal stroke events were 17 (11.2%) in the first group (1.9 events/100 patient-year) and 8 (3.5%) in the second group (0.5 events/100 patient-year) (*p* = 0.003). CV death events were 16 (10.5%) in the first group (1.8 events/100 patient-year) and 8 (3.5%) in the second group (0.5 events/100 patient-year) (*p* = 0.006) (Table 2). In addition to CV death, during the follow-up, 46 (12.2%) deaths due to non-CV causes occurred (1.9 events/100 patient-year), of which 30 (19.7%) in patients with SA < 3.5 g/dL (3.4 events/100 patient-year) and 16 (7.0%) in patients with SA ≥ 3.5 (1.0 events/100 patient-year) (*p* < 0.001). To further evaluate the relationship between SA and MACE, we divided the population into increasing tertiles of SA, each tertile consisted of 126 patients. Specifically, we observed more MACE in the tertile with lower SA levels followed by the second and third tertiles, in detail: 52 MACE in the first tertile, 28 in the second tertile and 14 in the third tertile, confirming the association between reduced SA levels and MACE (*p* < 0.001). Regarding hHF during follow-up, there were 112 (29.6%) hospitalizations in the whole study population (4.6 events/100 patient-year), of which 68 (44.7%) in the group with SA < 3.5 g/dL (7.7 events/100 patient-year) and 44 (19.5%) in the group with SA ≥ 3.5 g/dL (2.8 events/100 patient-year) (*p* < 0.001).

**Table 2:** Cardiovascular events and hHF in the study population according to clinical cut-off of serum albumin

	All population (n. 378)	Albumin < 3.5 g/dl (n. 152)	Albumin ≥ 3.5 g/dl (n. 226)	*p*
MACE, *n* (%)	94 (3.8)	60 (6.8)	34 (2.1)	< 0.001*
Nonfatal Stroke, *n* (%)	25 (1.0)	17 (1.9)	8 (0.5)	0.003*
NF Coronary events, *n* (%)	45 (1.8)	27 (3.1)	18 (1.1)	0.003*
CV mortality, *n* (%)	24 (0.9)	16 (1.8)	8 (0.5)	0.006*
Non CV Mortality, *n* (%)	46 (1.9)	30 (3.4)	16 (1.0)	< 0.001*
Total mortality, *n* (%)	70 (2.8)	46 (5.2)	24 (1.5)	< 0.001*
hHF, *n *(%)	112 (4.6)	68 (7.7)	44 (2.8)	< 0.001*

*performed by chi square test

Data described as number of patients (number of events per 100 patient-year)

*hHF* Heart failure hospitalizations, *MACE* major adverse cardiovascular events, *NF* Non-fatal, *CV* cardiovascular

**Fig. 2:**
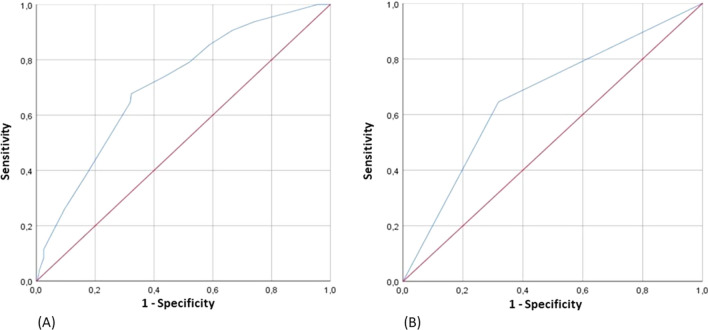
**A** ROC curves on MACE, according to SA as continuous variable. *AUC* area under the curve, *MACE* major cardiovascular adverse events, *ROC* receiver operating characteristic, *SA* serum albumin. **B** ROC curves on MACE, according to SA as dichotomous variable. *AUC* area under the curve, *MACE* major cardiovascular adverse events, *ROC* receiver operating characteristic, *SA* serum albumin

The accuracy of SA as a predictive value of the onset of MACE both as a continuous (Fig. 2A) and as dichotomous value (Fig. 2B) was evaluated by AUC. Figure 2A shows the ROC curve of SA as a continuous variable; notably, SA as a continuous variable has a greater discriminating power in predicting the development of MACE (AUC 0.708; standard error 0.030; 95% CI 0.650–0.766; *p* < 0.001), compared with SA as a dichotomous value (AUC 0.663; standard error 0.032; 95% CI 0.600–0.725; *p* < 0.001) (Fig. 2B).

### Cox regression analysis

Cox linear regression analysis shows a statistically significant association between MACE and age (considering 10 years’ variation), CKD, Ischaemic heart disease (IHD), systolic blood pressure (SBP) and UA > 6 mg/dL (Table 3). The variables significantly associated with the onset of MACE in the Cox univariate analysis were included in a multivariate analysis model to define the independent predictors of clinical outcome (Table 4). Of interest, albumin levels < 3.5 g/dL were associated with 3.1-fold increase in the risk of MACE, the presence of IHD and circulating UA levels > 6.0 mg/dL explained an increased risk of MACE by 2.3- and 2.2-fold, respectively. The presence of CKD also doubles the risk of MACE. Finally, a 10-year increase in age results in a 33% higher risk of MACE.The text continues here.

**Table 3:** Univariate Cox regression analysis on MACE

	HR	95% CI	*p*
Female gender, *yes/no*	1.471	0.183–11.820	0.717
Age, 10 years	1.296	1.021–1.824	0.013
IHD, *yes/no*	2.241	1.940–5.343	0.046
AF, *yes/no*	2.164	0.753–6.218	0.152
SAS, *yes/no*	1.015	0.522–1.972	0.965
CKD, *yes/no*	3.449	1.559–7.629	0.002
COPD, *yes/no*	2.449	0.923–6.499	0.072
NAFLD, *yes/no*	1.619	0.693–3.783	0.265
Obesity, *yes/no*	1.346	0.653–2.777	0.421
T2DM, *yes/no*	1.019	0.474–2.193	0.961
Alcohol, *yes/no*	1.400	0.518–3.781	0.507
Smokers, *yes/no*	1.397	0.659–2.962	0.383
SBP, *mmHg*	1.030	1.003–1.058	0.032
DBP, *mmHg*	0.981	0.938–1.025	0.387
HR, *bfm*	1.014	0.991–1.037	0.239
RR, *afm*	1.162	0.951–1.419	0.143
Hb, *g/dl*	1.156	0.865–1.544	0.327
Hct, %	1.048	0.960–1.145	0.293
K, *mmol/l*	1.345	0.596–3.038	0.476
HbA1c > 6.8%, *yes/no*	1.871	0.956–3.662	0.068
LDL, *mg/dl*	1.002	0.990–1.015	0.723
AST, *1 U/L*	1.006	0.983–1.029	0.622
ALT,* 1 U/L*	1.024	1.000–1.048	0.055
γ-GT, *1 U/L*	0.990	0.978–1.002	0.109
MNA, *1 pt*	0.955	0.897–1.016	0.141
Albumin < 3.5 g/dl, *yes/no*	6.177	2.895–13.182	< 0.001
NT-pro-BNP, *pg/ml*	1.000	1.000–1.001	0.286
UA > 6.0 mg/dl, *yes/no*	3.706	1.900–7.227	< 0.001
Iron deficiency, *yes/no*	2.875	1.081–7.649	0.034
hs-CRP, *mg/l*	0.797	0.642–0.990	0.040
NYHA class I, *yes/no*	2.035	0.061–68.111	0.692
NYHA class II, *yes/no*	6.435	0.644–64.263	0.113
NYHA class III, *yes/no*	1.706	0.171–16.982	0.649

*MACE* major adverse cardiovascular events, *IHD* ischemic heart disease, *AF* atrial fibrillation, *SAS* sleep apnea syndrome, *CKD* chronic kidney disease, *COPD* chronic obstructive pulmonary disease, *NAFLD* non-alcoholic fatty liver disease, *T2DM* type 2 diabetes mellitus, *SBP* systolic blood pressure, *DBP* diastolic blood pressure, *HR* heart rate, *RR* respiratory rate, *Hb* hemoglobin, *Hct* hematocrit, *K* Potassium, *HbA1c* glycated hemoglobin, *LDL* low-density lipoproteins, *AST* aspartate aminotransferase, *ALT* alanine aminotransferase, *γ-GT* gamma-glutamyltransferase, *MNA* mini nutritional assessment, *NT-pro-BNP* N-terminal pro-brain natriuretic peptide, *UA* uric acid, *hs-CRP* high-sensitivity C-reactive protein

**Table 4:** Multivariate stepwise Cox regression analysis on MACE

	HR	CI 95%	*p*
Albumin < 3.5 g/dl, yes/no	3.114	1.899–5.106	< 0.001
IHD, yes/no	2.264	1.311–3.909	0.003
UA > 6.0 mg/dl, yes/no	2.183	1.275–3.737	0.004
CKD, yes/no	2.050	1.263–3.327	0.004
Age, 10 years increase	1.336	1.055–1.692	0.016

*MACE* major adverse cardiovascular events, *IHD* ischemic heart disease, *UA* uric acid, *CKD* chronic kidney disease

## Discussion

This study, conducted in a population of middle-age outpatients with HF (both HFrEF and HFmrEF/HFpEF, NYHA class II and III) with several comorbidities, showed that there is an association between low SA levels and incidence of MACE during a median follow-up of 6.1 years. Specifically, SA values < 3.5 g/dl increased the risk of MACE by 3.1-fold. This association also persisted after adjustment of other demographic and clinical variables, including age, sex, concomitant cardiovascular disease, nutritional status, liver and kidney function. The analysis of the processed ROC curve and measurement of relative AUC also demonstrated the accuracy of SA levels as predictor of MACE occurrence. These results are clinically relevant because albumin represents a routinely dosed analyte in daily clinical practice for HF patients with several comorbidities.

SA value < 3.5 g/dL has already been described as a risk factor for the development of MACE in both inpatients and outpatients [40, 41]. In fact, in the general population, hypoalbuminemia is a powerful and independent predictor of all-cause mortality and CV mortality [42, 43]. In addition, several studies have shown that hypoalbuminemia predicts the occurrence of HF in particular patients’ settings, such as CKD, elderly and diabetic patients independently of traditional CV risk factors and CV and non-CV comorbidities [38, 39]. In recent years, the prognostic role of low SA value in patients with HF has been established, but with different study population and methodology. Indeed, several studies have demonstrated a close association between reduced SA levels and poor prognosis in both acute and chronic HF [40, 42]. However, these studies considered mainly hospitalized or elderly subjects during a short follow-up, in addition patients showed frequently malnutrition, with a reduction in the functional reserve for a greater burden of comorbidities.

According with this, in a retrospective cohort study including 8,246 patients hospitalized for acute HF, hypoalbuminemia was an important predictor of 30-day and 1-year mortality in this setting, regardless of the HF phenotype, both HFrEF and HFpEF. Moreover, SA levels < 3.4 g/dl were associated with a two-fold increase in mortality risk at 1 year, in elderly patients hospitalized for acute HF. In this study more than 50% of the population showed SA levels < 3.4 g/dL, this could be explained by the higher number of comorbidities, especially liver and kidney disease, and by hospitalization that results in a major inflammatory burden, all conditions that may negatively affect circulating albumin levels [21]. Considering this, it is not surprising that in the sub-analysis of the CHARM study, after adjustment for confounding factors, albumin has lost its prognostic role during a median follow-up of 3.2 years, in fact in the multivariate model also parameters of liver dysfunction were included [7]. Furthermore, in the COAPT study, in patients with chronic HF and functional mitral insufficiency, having serum albumin levels < 4 g/dl was associated with reduced survival over a 4-year follow-up, but was not associated with an increased incidence of hospitalization [28].

In addition, an observational study of 5779 chronic HF patients with a mean age of 75 years confirmed the prognostic role of SA. Indeed, SA levels < 3.8 g/dl were associated with an increased risk of mortality (HR: 5.74, 95% CI: 4.08–8.07, *p* < 0.001) during a mean follow-up of 576 days. Moreover, in the study, SA levels at follow-up were available in 77% of enrolled patients, and it was seen that a decrease in albumin on follow-up was an independent predictor of increased mortality (HR: 2.58, 95% CI: 2.12–3.14, *p* < 0.001); confirming the prognostic role of SA in patients with chronic HF [29].

Our study has been conducted in middle-aged outpatients, with a median follow-up of 6.1 years excluding patients with liver dysfunction and malnutrition, well-known confounders that may affect SA levels and MACE occurrence. Of interest, in our study SA levels did not lose their predictive power even after correction for liver and kidney function, inflammation, and nutritional status. In addition, ROC curves analysis showed a predictive accuracy of SA levels both as continuous (AUC 0.708) and dichotomous (AUC 0.663) value for MACE occurrence. This result is consistent with previous data from our group reporting a high prevalence of SA < 3.5 g/dL in type 2 diabetes mellitus (T2DM) patients with a significant association with poor survival and enhanced occurrence of venous and arterial thrombosis [40]. In fact, hypoalbuminemia as a marker of underlying comorbidities, malnutrition, cachexia, and inflammatory state, may justify a poor clinical prognosis [6]. However, in the present study circulating albumin levels were mainly justified by hs-CRP, indicating that chronic low grade inflammation could represent the main predictor of SA levels.

Of interest, in our analysis IHD, UA and CKD doubled the risk of MACE in the study population and age as 10 years increase augmented the risk of 33%, thus confirming the prognostic value of these factors in particular in HF patients [44–46]. Thus, it is appropriate to ask whether hypoalbuminemia is only an epiphenomenon, a marker of comorbidities burden and inflammation, or it represents a causal factor for MACE. Of interest, besides to be the most represented protein in blood, albumin performs several physiological functions; in fact, it is primarily responsible for oncotic blood pressure and it can inhibit coagulation activation through modulation of hepatic biosynthesis of coagulation factors or heparin-like activity [47]. In addition, albumin exerts antiplatelet activity by blocking thromboxane A2 activity or via an oxidative stress-mediated mechanism [48]. In fact, hypoalbuminemia confirms its prognostic role in different clinic al settings, it increases the risk of MACE in acute and chronic HF, but also in hospitalized patients regardless of the cause of hospitalization and in T2DM. Furthermore, in our work, the prognostic role of circulating albumin levels is confirmed for long-term follow-up, even after correction for known confounding factors, in particular for clinical conditions that may result in hypoalbuminemia such as nutritional status, inflammation, liver and kidney disease [40, 41]. Finally, we observed that patients with SA values < 3.5 g/dl showed an increased risk for hHF compared to patients with higher SA values.

These results are in agreement with previous evidence demonstrating that reduced SA levels increase the risk of HF onset and progression. In fact, hypoalbuminemia, by reducing the colloid-osmotic pressure of the bloodstream, can promote pulmonary and systemic congestion with a consequent worsening of heart failure [11, 12]. According with this, as a future perspective, the potential prognostic role of serum albumin on risk on a combined endpoint of non-fatal events, and additionally on hHF and all-cause mortality, could be evaluated.

The present study has also several limitations; at first, it is a retrospective observational study. Another important limitation is represented by the imbalance of patients’ number according to two groups, the low percentage of women and the relatively small sample size with a long duration of enrollment. In addition, our study measured SA levels only at baseline and did not assess whether increasing SA levels could result in an improved prognosis and eventually a reduction in MACE; this issue deserves further investigations. This suggests a potential role of SA as a biomarker able to identify patients with chronic HF at high risk of developing MACE even in the absence of malnutrition and inflammation, in this context it is not possible to exclude a direct role of albumin in CV risk. According with this, it’s important to remark the potential negative impact of hypoalbuminemia on efficacy of antithrombotic and antiplatelet drugs, further increasing the risk of MACE [40, 41].

Considering the results of our study, it seems important to include SA levels among the variables considered in the scores used to stratify the risk of patients with HF such as the MECKI score for HFrEF, and to the variables considered in the MAGGIC meta-analysis for HF in the various phenotypes [49, 50].

## Conclusion

This study, conducted on outpatients with HF and several comorbidities, showed that there is an association between lower SA levels and MACE occurrence during a long-term follow-up. In particular, a value < 3.5 g/dL of SA levels was associated with a 3.1-fold increased risk. Notably, this association persists even after adjustment for confounding factors such as age, inflammation, nutritional status, liver and kidney function. Present data suggest the potential usefulness of SA as prognostic factor for incident MACE and hHF in outpatients with HF during a long-term follow-up, in particular for a value < 3.5 g/dL and it may be helpful to identify patients with a lower response to antithrombotic drugs. Of interest, in the study population, the group with SA < 3.5 g/dl had a higher total mortality than the group with SA ≥ 3.5 g/dl. In conclusion, a considerable proportion of patients with chronic HF exhibits low SA levels and show a higher risk of MACE, hHF and total mortality.

### Supplementary Information

Below is the link to the electronic supplementary material.

Supplementary file1 (TIFF 900 kb)

Supplementary file2 (DOCX 20 kb)

## Data Availability

Not applicable.

## Code availability

Not available.
