# Supplementary figures and images for: Prognostic role of serum albumin levels in patients with chronic heart failure
Source: Intern Emerg Med. 2024 May 22;19(5):1323–33. doi: 10.1007/s11739-024-03612-9 (PMC11364577; doi:10.1007/s11739-024-03612-9)

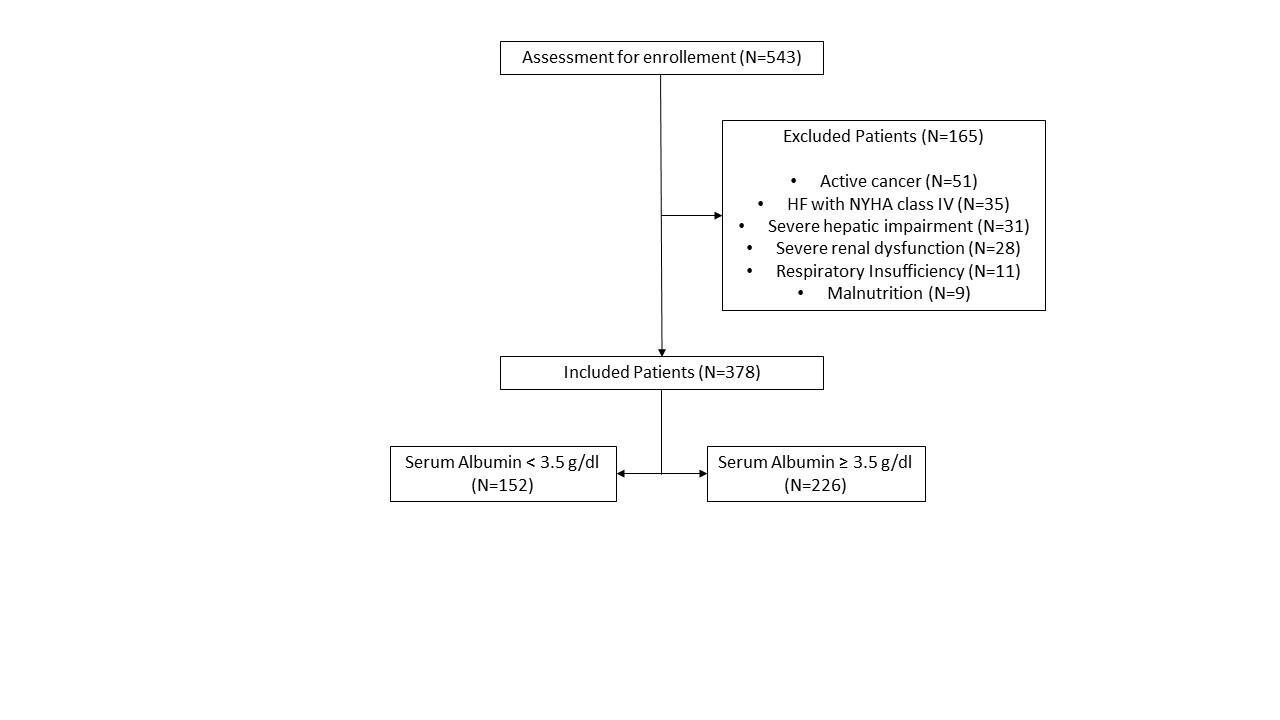

Supplement: Supplementary file 1 — Supplementary file1 (TIFF 900 kb) [file 11739_2024_3612_MOESM1_ESM.tiff]
