# Supplementary material for: Prognostic role of serum albumin levels in patients with chronic heart failure
Source: Intern Emerg Med. 2024 May 22;19(5):1323–33. doi: 10.1007/s11739-024-03612-9 (PMC11364577; doi:10.1007/s11739-024-03612-9)
Supplement: Supplementary file 2 — Supplementary file2 (DOCX 20 kb) [file 11739_2024_3612_MOESM2_ESM.docx]

**Supplementary table 1. Simple linear correlation analysis between SA as continuous value and SA as dichotomous value and different covariates in the study population.**

|  | **SA,** *as continuous value* | **SA,** *as dichotomous value* |
| --- | --- | --- |
|  | r/p | r/p |
| **Female Gender,** *yes/no* | 0.080/0.175 | 0.075/0.171 |
| **Age,** *years* | -0.038/0.374 | -0.049/0.220 |
| **CKD,** *yes/no* | -0.035/0.422 | -0.057/0.153 |
| **NAFLD,** *yes/no* | -0.206/<0.001 | -0.114/0.004 |
| **Obesity,** *yes/no* | -0.023/0.592 | -0.029/0.459 |
| **T2DM,** *yes/no* | -0.033/0.558 | -0.003/0.953 |
| **HOMA,** *pt* | -0.047/0.401 | -0.003/0.953 |
| **NT-pro-BNP,** *pg/ml* | -0.063/0.140 | -0.042/0.285 |
| **Uricemia,** *mg/dl* | -0.026/0.545 | -0.001/0.978 |
| **hs-CRP,** *mg/l* | -0.398/<0.001 | -0.513/<0.001 |
| **Microalbuminuria,** *mg/l* | -0.109/0.015 | -0.039/0.004 |
| **MNA,** *pt* | 0.189/<0.001 | 0.213/<0.001 |
| **Dislipidemia,** *yes/no* | -0.080/0.063 | -0.118/0.073 |

**Abbreviations: SA,** serum albumin; **CKD,** Chronic kidney disease; **NAFLD,** Non-alcoholic fatty liver disease, **T2DM,** Type 2 Diabetes mellitus; **HOMA**, homeostatic model assessment; **hs-CRP**, highly sensitive c-reactive protein; **MNA:** Mini-Nutritional Assessment.

**Supplementary table 2. Stepwise multivariate linear analysis between SA as continuous value and SA as dichotomous value and different covariates in the study population.**

| **SA, as continuous value** | **R^2^ partial** | **R^2^ total** | **p** | **SA as dichotomous value** | **R^2^ partial** | **R^2^ total** | **p** |
| --- | --- | --- | --- | --- | --- | --- | --- |
| **hs-CRP,** *mg/l* | 24.8 % | 24.8 % | <0.001 | **hs-CRP,** *mg/l* | 36.5 % | 36.5% | <0.001 |
| **NAFLD,** *yes/no* | 5.5 % | 30.3 % | <0.001 | **MNA,**  *yes/no* | 4.7 % | 41.2 % | <0.001 |
| **MNA,**  *yes/no* | 3.1 % | 33.4 % | <0.001 | **Microalbuminuria,** *mg/l* | 1.5 % | 42.7 % | 0.001 |
| **Microalbuminuria,** *mg/l* | 1.9 % | 35.3 % | 0.03 | **NAFLD,** *yes/no* | 0.9 % | 43.6 % | 0.007 |

**Abbreviations: SA,** serum albumin; **hs-CRP**, highly sensitive c-reactive protein; **NAFLD,** Non-alcoholic fatty liver disease, **MNA:** Mini-Nutritional Assessment.
